# PTBP3 Induced Inhibition of Differentiation of Gastric Cancer Cells Through Alternative Splicing of Id1

**DOI:** 10.3389/fonc.2020.01477

**Published:** 2020-08-18

**Authors:** Bin Chen, Weixia Chen, Xiaoyan Mu, Liyan Yang, Xiangyu Gu, Aiguang Zhao, Xin Liang, Jianwen Liu

**Affiliations:** ^1^Department of Oncology, Longhua Hospital, Shanghai University of Traditional Chinese Medicine, Shanghai, China; ^2^Department of Oncology, Putuo Hospital, Shanghai University of Traditional Chinese Medicine, Shanghai, China; ^3^State Key Laboratory of Bioreactor Engineering and Shanghai Key Laboratory of New Drug Design, School of Pharmacy, East China University of Science and Technology, Shanghai, China

**Keywords:** alternative splicing, gastric cancer, PTBP3, Id1, Hes1

## Abstract

Overexpression of PTBP3, a factor involved in alternative splicing, may inhibit the differentiation of leukemia cells. However, its role in gastric cancer differentiation and the specific pathways involved are unclear. In this study, we found that PTBP3 was upregulated in the poorly differentiated gastric cancer tissues. Patients with high levels of PTBP3 expression had significantly shorter survival than those with low PTBP3 expression. In gastric cancer cells, the regulatory effect of PTBP3 on alternative splicing of the *Id1* gene was investigated. Following sodium butyrate-induced differentiation of MKN45 cells, the expression of Id1a decreased, but the expression of Id1b increased. RNA interference and overexpression experiments showed that PTBP3 upregulated Id1a expression and downregulated Id1b expression. RNA immunoprecipitation (RIP) assays indicated PTBP3 could interact with Id1. UV cross-linking assays indicated that PTBP3 interacted with the CU rich region of the *Id1* gene. Two-hybrid experiments and a gel mobility shift assays found that Id1b had a more potent affinity for Hes1 than Id1a. Chromatin immunoprecipitation (ChIP) assays verified the association of Hes1 and the promoter of *PTBP3* gene. Luciferase assays revealed that Hes1 bound the N-box sequence in the *PTBP3* promoter. After silencing or overexpression of Hes1, PTBP3 protein expression remained unchanged. Thus, the loss of feedback regulation among PTBP3, Id1, and Hes1 in gastric cancer cells may be one of the causes of inhibited differentiation and malignant proliferation of these cells.

## Introduction

Alternative splicing of pre-mRNA is a common phenomenon in eukaryotes and an important way to regulate gene expression. In humans, alternative splicing is observed in about 92–94% of genes (1). Abnormal alternative splicing of some genes has been shown in some cancers (2–5). Alternative splicing can be regulated by the SR protein family and the hnRNP protein family (6, 7).

PTBP3 is a member of the hnRNP family. Our previous study indicated that the expression of PTBP3 in gastric cancer was higher than in normal gastric mucosa, and it inhibited the differentiation of MKN45 cells and promoted their proliferation (8). These findings suggested that PTBP3 plays a crucial role in the regulation of gastric cancer cell differentiation, but the specific mechanism is still poorly understood.

Basic helix-loop-helix (bHLH) proteins can be divided into two subgroups: A and B. bHLH proteins of subgroup A are ubiquitously expressed transcription factors, such as E2A and HEB; bHLH proteins of subgroup B have tissue-specificity, such as Hes1, MyoD, dHAND, and HASH-1. These bHLH proteins can form homodimers and heterodimers with each other to recognize the E-box or N-box in a specific gene, which then regulates the differentiation and proliferation of cells (9). Hes1 expression has been found in several gastric cancer cell lines (10). Hes1 is a transcriptional repressor that inhibits the differentiation of cancer cells (11), playing an essential role in the pathogenesis of cancers.

Id1 is a member of the helix loop helix (HLH) trans-acting factor family. It has no DNA binding region and can form heterodimers with bHLH to block its DNA binding, which may affect gene expression, inhibit cell differentiation, and promote cell proliferation (12, 13). In addition, overexpression of Id1 *in vitro* inhibits the differentiation of a variety of types of cells (14, 15). High expression of Id1 has been confirmed in gastric cancer tissues and cell lines, suggesting that it plays a critical role in the occurrence of gastric cancer (16, 17). Id1 has two alternatively spliced mRNA isoforms, arising from alternative retention of the single intron in the Id1 gene. This leads to two protein isoforms Id1a and Id1b, which have unique 13 and 7 amino acid C-terminal tails, respectively (18). Id1a promotes cell proliferation, but Id1b inhibits cell proliferation and facilitates cell renewal, suggesting opposite biological functions of Id1a and Id1b (19, 20). Whether the transcriptional isoforms of Id1 (Id1a and Id1b) are generated by PTBP3 through alternative splicing, whether PTBP3-induced inhibition of cell differentiation is related to PTBP3-regulated alternative splicing of Id1 as well as the binding of Id1 to bHLH are still unclear.

In this study, we found that PTBP3 can regulate the expression of Id1a and Id1b isoforms via alternative splicing. Furthermore, Id1a and Id1b may differentially bind to Hes1 to inhibit Hes1 activity, which may be related to PTBP3-induced inhibition of cellular differentiation. Our findings contributed to understanding the relationship between alternative splicing and the occurrence and development of gastric cancer.

## Materials and Methods

### Tissue Microarray and Immunohistochemistry

The human gastric cancer tissue microarray (HStmA180SUR07, Shanhai Xinchao, China) consisting of 90 gastric adenocarcinoma tissue samples and 90 matched adjacent normal gastric mucosa tissue samples. Immunohistochemistry were performed as previously described (8).

### cDNA Microarray and Quantitative Real-Time PCR

The human gastric cancer cDNA microarray (HStmA060CS01, Shanhai Xinchao, China), which contains 30 gastric adenocarcinoma tissue samples and 30 matched adjacent normal gastric mucosa tissue samples. Total RNA was isolated by TRIZOL™ reagent (Invitrogen, Carlsbad, CA, USA) and then reverse transcribed using the Reverse Transcription System (Promega, Madison, WI, USA) according to the manufacture's instruction. Quantitative Real-time PCR was carried out on a Bio-Rad Real-Time PCR system. Gene expression was quantified as the level of indicated genes relative to that of GAPDH.

The sequences of the Real-time primers were as follows:

PTBP3 sense: 5′-TCTCCGTGCCTTCAGTCAGC-3′,
and anti-sense: 5′-GCTTTACGCATCGTCACGC-3′;
GAPDH sense: 5′-GGTCGGAGTCAACGGATTTG-3′,
anti-sense: 5′-ATGAGCCCCAGCCTTCTCCAT-3′.

### Cell Culture and Induction of Differentiation

Gastric cancer cell lines MKN45, and HGC27 were purchased from the Type Culture Collection of the Chinese Academy of Sciences (Shanghai, China). For drug-induced cell differentiation, NaBU (Sigma, St. Louis, MO, USA) was dissolved in PBS, adjusted to pH 7.2 with NaOH, adjusted to a concentration of 3 mM, and filter sterilized.

### Total RNA Extraction

Total RNA was extracted using TRIZOL™ reagent (Invitrogen, Carlsbad, CA, USA) and was reverse transcribed with (dT)n primers and Superscript III Reverse transcriptase (TaKaRa Biotechnology, Dalian, China). The following primer sequences were used:

Id1a sense: 5′-TCAACGGCGAGATCAGC-3′,
anti-sense: 5′-TCGGTCTTGTTCTCCCTCA-3′;
Id1b sense: 5′-TGCCTAAGGAGCCTGGAA-3′,
anti-sense: 5′-CCGCCTGTGAAAACGAGA-3′.

### Western Blot Analysis and Antibodies

Total protein extracts were obtained as previously described (21). The following primary antibodies were used: PTBP3 (Thermo Fisher Scientific, USA), Hes1 (Sigma, St. Louis, MO, USA), b-actin (Sigma, St. Louis, MO, USA), Id1a (against residues 142–153 [EAACVPADDRIL], obtained from Shanghai Youke Biotechnology, working concentration: 10 ug/ml), Id1b (against residues 139–149 [LTAEVRSRSDH], obtained from Shanghai Youke Biotechnology, working concentration:10 ug/ml).

### Chromatin Immunoprecipitation (ChIP) Assay

MKN45 cells were cross-linked with 1% formaldehyde for 10 min. ChIP assays were performed using the EZ ChIP Kit (Millipore, Bedford, MA, USA), according to the manufacturer's instructions. Anti-rat IgG were used as controls. The primer sequences were as follows:

PTBP3 primer 1 sense: 5′-AGTGCTTGAATTTTCCAAAATGAGC-3′,
anti-sense: 5′-GCCAGATGGAAATGTAGAATATCAA-3;
PTBP3 primer 2 sense: 5′-GGGACTGAGGTTAGATACAG-3,
anti-sense: 5′-ACAAGAAAATCAGGTTCCAG-3;
PTBP3 primer 3 sense: 5′-AGAGGCTGGTCCCTGGACGTGTCAT-3,
anti-sense: 5′-CGGGCCTGCCAGGTTTGAAT-3;
PTBP3 primer 4 sense: 5′-GGGGAGTTGCTGTTTACCGG-3,
anti-sense: 5′-AGCACAAGTCGGGAGACCTC-3.

### RNA Immunoprecipitation (RIP) Assay

RIP experiments were performed with the Magna RIP RNA-Binding protein immunoprecipitation kit (Millipore, Bedford, MA, USA) according to the manufacturer's instructions. The collected RNAs were subjected to RT–qPCR analysis to quantify the enrichment of PTBP3. Anti-rat IgG were used as controls. The primer sequences were as follows:

Id1 sense: 5′-CATGCGTTCCTGCGGACGAT-3,
anti-sense: 5′-CCGATCGGTCTTGTTCTCCC-3.

### Vector Constructs and Dual-Luciferase Reporter Gene Assays

Cells were transfected using Oligofectamine reagent Lipofectamine™ 2000 (Invitrogen, Carlsbad, CA, USA). The coding region of cDNA was cloned into the pcDNA 3.1(+) vector, and the constructs were verified by DNA sequencing. The siRNA sequence of PTBP3 was chemically synthesized. The primer sequences were as follows:

siRNA (PTBP3): 5′-GAGUGAAGAUUAUGUUUAATT-3′;
pcDNA (PTBP3) sense: 5′-CAAGCTTGACACCAGGGGTCTGGACTTA-3′,
anti-sense: 5′-CG GGATCCTATGGTCCAGTTTTAGGAGA-3′;
pGL3-basic (PTBP3) sense: 5′-CCTCGAGTGAGGCAGGAGAATCACTTGAAC-3′, anti-sense: 5′-CAAGCTTTA ACCGCGAGCAGAGGAAGCAG-3′;
pcDNA (Hes1) sense: 5′-TAGCTAGCGG GATCACACAGGATC-3′,
anti-sense: 5′-CCGCTCGAGAAAAGCCTTTACTTT T-3′;
siRNA (Hes1): 5′-CAACACGACACCGGAUAAA-3′;
pcDNA (Id1a) sense: 5′-CCCAAGCTTGCCACCATGAAAGTCGCCAGTGGCAGCACCG-3′,
anti-sense:5′-CCGGAATTCTCAGCGACACAAGATGCGATCGTCC-3′;
pcDNA (Id1b) sense:
5′-CCCAAGCTTGCCACCATGAAAGTCGCCAGTGGCAGCAC CG-3′,
anti-sense:5′-CCGGAATTCCTAGTGGTCGGATCTGGATCTCACCTCGGCCGTCAGGGCGCTGATCTC-3′.

Dual-luciferase reporter gene assays were performed using the Dual-Luciferase Reporter Assay System (Promega, Madison, WI, USA). Cell lysates were assayed for luciferase activity following the manufacturer's protocol (Promega, Madison, WI, USA), using a Centro XS3 LB960 microplate luminometer (Berthold Technologies, Bad Wildbad, Germany). Renilla luciferase activity was normalized to firefly luciferase activity.

### UV Cross-Linking Assay

The templates for *in vitro* RNA synthesis were derived from a minigene containing an Id1 intronic fragment downstream of the T7 promoter. The RNA probes were synthesized by T7 RNA polymerase in the presence of [α-^32^P]UTP using a Transcription T7 Kit (Takara Biotechnology, Dalian, China). UV cross-linking was performed using 2 pmol ^32^P- labeled RNA and 50 μg of protein extracts. Proteins and RNA were incubated at 30°C for 30 min in 25 μL of binding buffer (20 mM Hepes pH 7.8, 50 ng yeast tRNA, 25 mM KCl, 2 mM MgCl_2_, 3.8% glycerol, 0.1 mM EDTA, 2 mM DTT, 1 mM ATP). Reaction mixtures were irradiated with 254 nm UV light for 10 min on ice. The residual RNA was degraded by 100 U/μL RNase A and RNase T1 (Takara Biotechnology, Dalian, China). Then, the samples were denatured and immunoprecipitated with anti-PTBP3. The samples were subject to SDS-PAGE followed by autoradiography. Sizes were determined using prestained markers (Takara Biotechnology, Dalian, China).

### Mammalian Two-Hybrid Analysis

Mammalian two-hybrid analyses were performed using a commercially available mammalian two-hybrid system (Promega, Madison, WI, USA), which includes the pACT, pBIND and pG5luc plasmids. The pG5luc reporter plasmid contains five GAL4-binding sites upstream of a minimal TATA box that precedes the firefly luciferase gene. The coding sequences of proteins were also generated by PCR, and cloned into pACT and pBIND at the BamHI/SalI sites. The nucleotide sequence of the inserts was verified by sequence analysis, and expression of all proteins was verified by Western blot analysis.

The luciferase assays were performed according to the manufacturer's recommendation. Approximately 48 h after each transfection, the luciferase activity was detected and normalized by Renilla activity.

### Electrophoretic Mobility Shift Assay

Using Lipofectamine™ 2000 (Invitrogen, Carlsbad, CA, USA), pcDNA (Hes1), pcDNA (Id1a), or pcDNA (Id1b) was transfected into gastric cells. After 48 h, the cells were harvested in lysis buffer, and DNA binding reactions were carried out in a total volume of 25 μL. Increasing amounts of cell extract from Id1a or Id1b plasmid-transfected cells were mixed with extracts from Hes1-transfected cells for 10 min at room temperature, after which a labeled N-box oligonucleotide probe was added for a further 15 min at room temperature. In order to increase the specific binding activity, we designed the probe containing the canonical N-box (5′-CACAAG-3′). Cell extracts from untransfected cells were used as a negative control. The binding buffer consists of 20 mM HEPES (pH 7.5), 5 μg/mL aprotinin, 50 mM KCl, 1 mM dithiothreitol, 1 mM EDTA, 4% glycerol, 1 μg of dl-dC. The binding reactions were then subjected to electrophoresis in a 6% polyacrylamide gel. The gels were dried, and the labeled complexes were detected by autoradiography.

### Xenotransplantation and Analysis of Tumors

All animal experiments were approved by the Animal Ethics Committee of Longhua Hospital. Approximately 1.0 × 10^7^ MKN45-pSliencer and MKN45-PTBP3 knockdown stable cells in 100 μL serum-free medium were implanted in the subcutaneous tissue of the right abdominal wall of female severe combined immunodeficient mice (7–8 week old, one tumor per mouse, *n* = 6 mice per group). Mice were sacrificed after 6 weeks, and tumors were collected. Samples of each tumor were snap-frozen in liquid nitrogen and then processed for further histologic analysis.

### Statistical Analysis

Statistical analysis was performed using SPSS 18.0 software. Variables were expressed as means ± SD. Comparisons between Kaplan-Meier curves were performed using the long-rank test. Correlations were determined by Pearson correlation. All other comparisons were analyzed by unpaired two-tailed Student's *t*-test. *P* < 0.05 were considered statistically significant.

## Results

### Increased Expression of PTBP3 Is Positively Associated With Poor Differentiation and Poor Prognosis in Gastric Cancer

**Figure 1:**
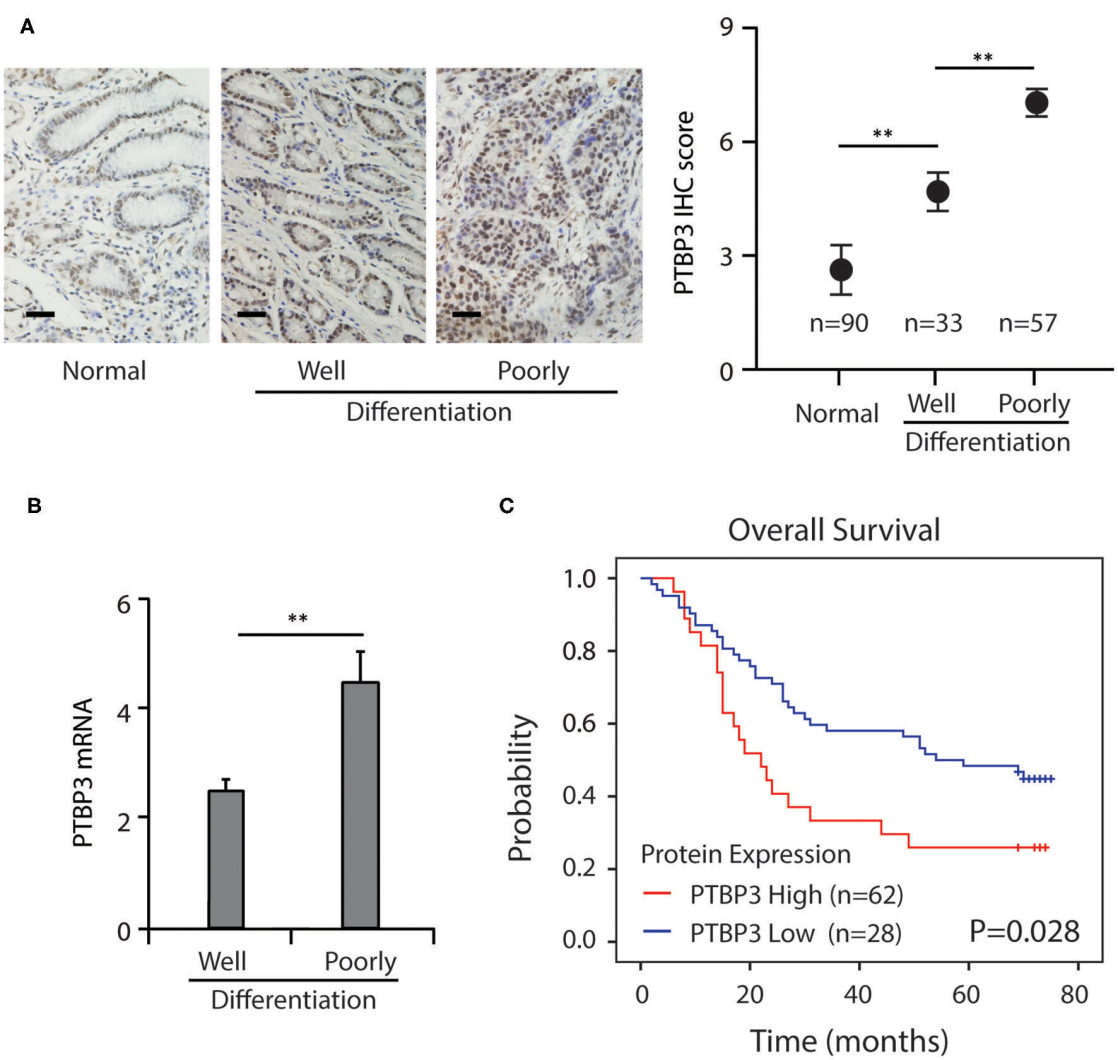
Increased PTBP3 expression was associated with poor survival in gastric cancer. **(A)** Representative images and quantitative IHC analysis of PTBP3 expression in normal gastric mucosa tissue, well and poorly-differentiation of gastric cancer tissue. Scale bar, 50 μm. **(B)** PTBP3 mRNA levels (log2 intensity) in well and poorly-differentiation of gastric cancer tissue. **(C)** Kaplan–Meier analysis of overall survival in patients with variable PTBP3 expression. ^*^P < 0.01.

Our previous study has shown that in gastric cancer tissues, PTBP3 protein was overexpressed (8). To further confirm these results and clarify the clinical significance of PTBP3, we first used immunohistochemistry to screen the expression of PTBP3 in adjacent normal tissues, well and poorly-differentiation of gastric cancer tissues by immunohistochemistry. Immunohistochemistry analysis showed that PTBP3 was up-regulated in the poorly-differentiation of gastric cancer tissues (Figure 1A). After testing the mRNA expression of PTBP3 in tissue samples from gastric cancer patients, we found PTBP3 level in the poorly-differentiation of gastric cancer tissues were significantly higher (Figure 1B). Kaplan–Meier survival analysis showed that gastric cancer patients with high PTBP3 expression had a worse outcome than those with low PTBP3 expression (*P* = 0.028, Figure 1C). These results imply that PTBP3 may play an important role in the regulation of gastric cancer differentiation.

### Influence of NaBU-Induced Differentiation of Gastric Cancer Cells on the Expression of Id1a, Id1b, PTBP3, and Hes1

**Figure 2:**
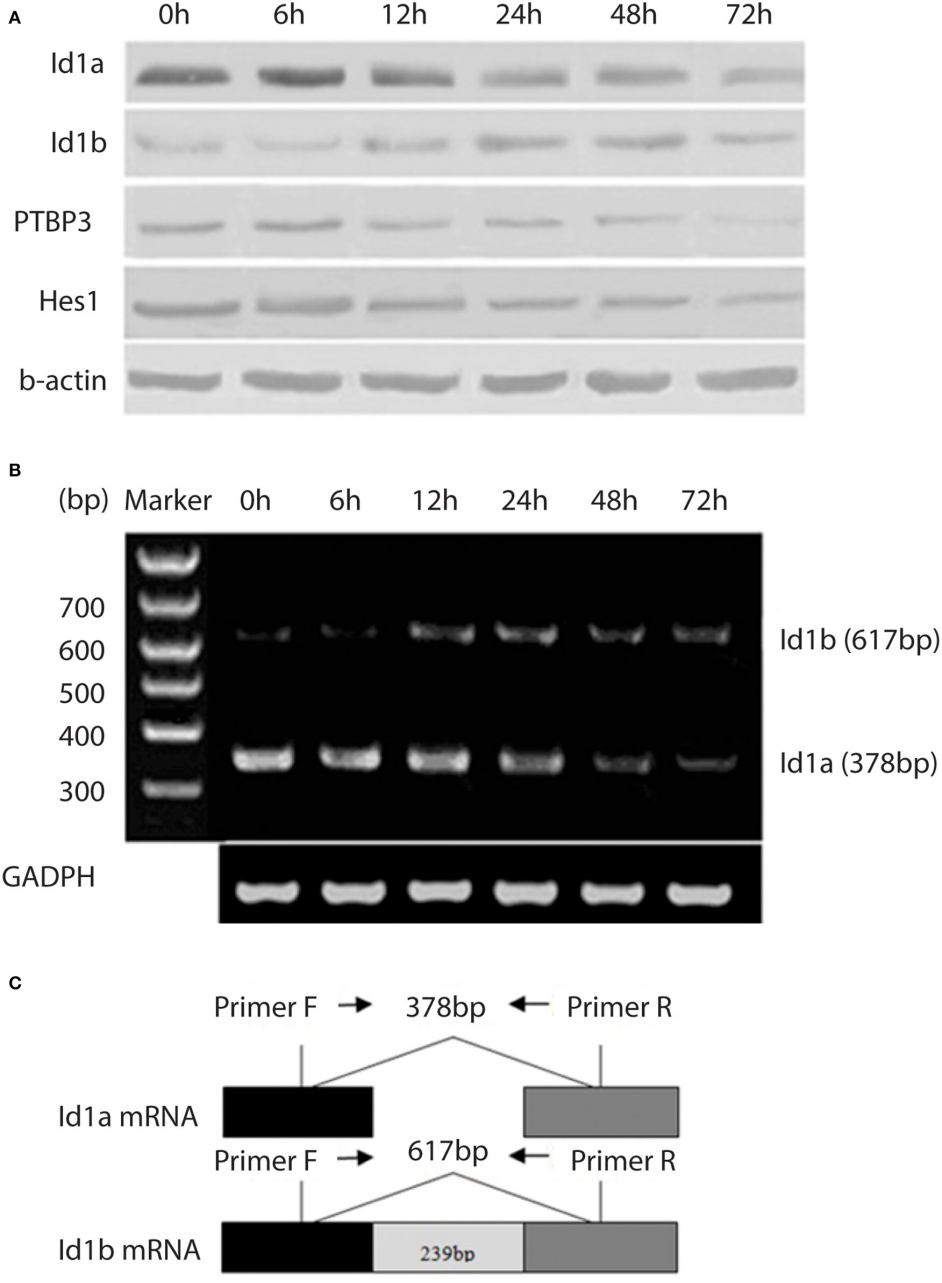
Expression of Id1a, Id1b, PTBP3, and Hes1 in the differentiating gastric cancer cells. **(A)** Western blot analysis of Id1a, Id1b, PTBP3, and Hes1 expressed in NaBU-treated MKN45 cells. The cells were treated with 3 mM NaBU for 6–72 h. **(B)** RT-PCR was performed to display the relative levels of Id1a and Id1b mRNAs in NaBU-treated MKN45 cells. **(C)** The map showed the primer sites for RT-PCR. Id1a and Id1b proteins differ at the C-terminus due to differential splicing of the 239 bp intron (sequence from NCBI).

NaBU (sodiumbutyrate) is a recognized differentiation inducer, which can induce the differentiation of many kinds of tumor cells, such as gastric cancer cells (22). To investigate changes in the expressions of PTBP3, Id1a, Id1b, and Hes1 in the differentiation of gastric cancer cells, NaBU was used as a differentiation-inducing agent in the MKN45 gastric cancer cell lines. NaBU at 3 mM inhibited cell growth to a certain extent, and these cells showed common characteristics of differentiation, such as reduced nucleus/cytoplasm (N/C) ratio and regular nuclei. Moreover, Id1a expression was downregulated over time, accompanied by upregulated expression of Id1b (Figure 2A). Changes in the mRNA expressions of Id1b and Id1a were similar to their protein expressions (Figures 2B,C). The protein expressions of PTBP3 and Hes1 were downregulated over time. These findings suggest that the expression of these genes changes during the induced differentiation of gastric cancer cells.

### PTBP3 Upregulates Id1a Expression and Downregulates Id1b Expression Through Binding to the CU-Rich Region of Id1 Intron

**Figure 3:**
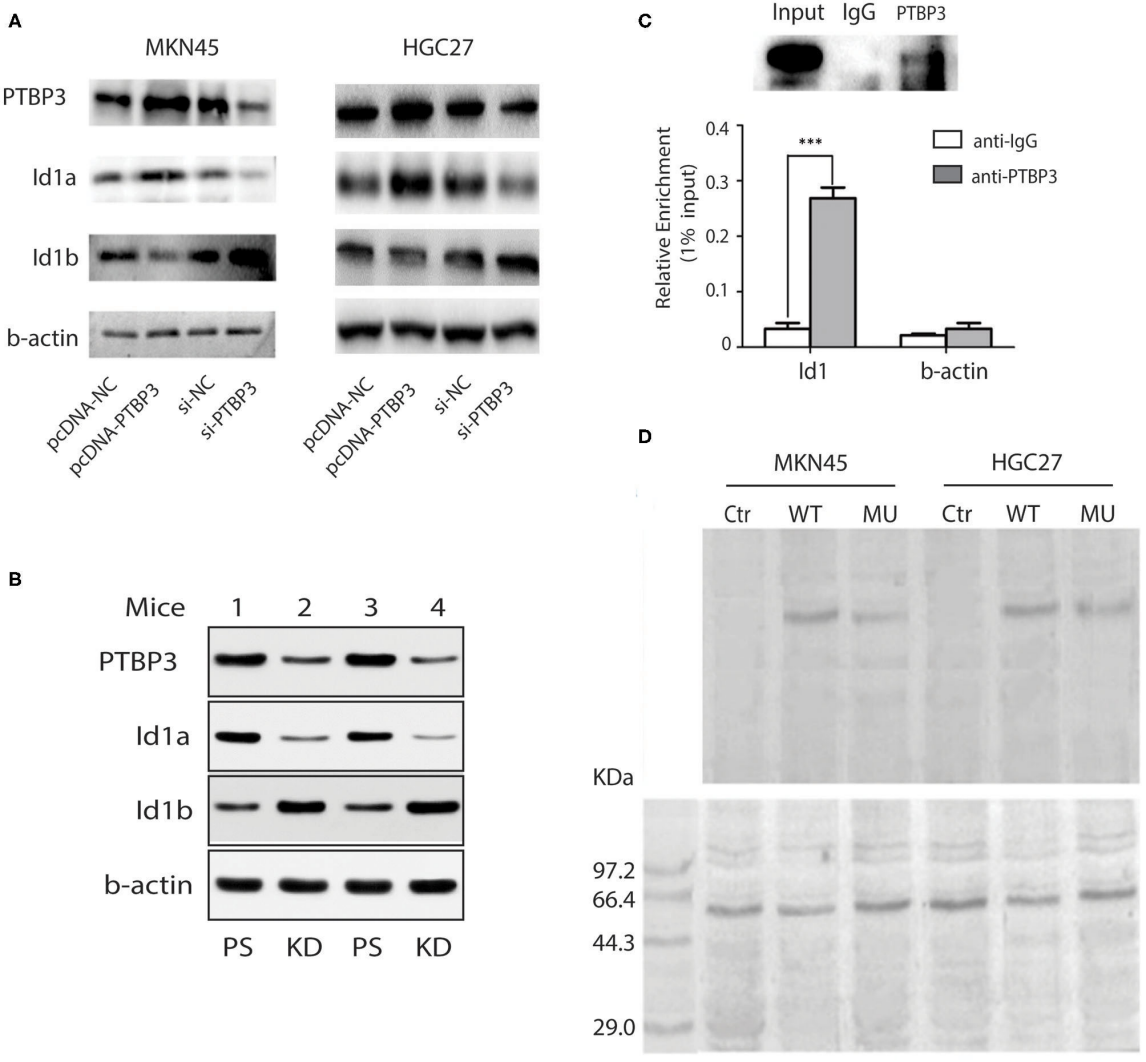
PTBP3 modulates Id1 alternative splicing via binding to its intronic region. **(A)** Immunoblots showed Id1a and Id1b protein expression levels after altering the PTBP3 expression level in two gastric cancer cells. **(B)** Western blot analysis of Id1a, Id1b, and PTBP3 expressed in mouse tumors from different PTBP3 knockdown and pSliencer vector-transfected mice. **(C)** RIP experiments were performed using the PTBP3 antibody to immunoprecipitate (IP) in total-cell extracts of MKN45 cells, and relative enrichment was determined by reverse transcription PCR (upper panel) and quantitative real-time PCR (lower panel). The results are expressed as the mean ± S.D. of three independent experiments. ***P < 0.001. **(D)** UV cross-linking was performed using 2 pmol 32P-labeled RNA and 50 μg of protein extracts. The immunoprecipitates were subjected to SDS–PAGE followed by autoradiography (upper panel), and the samples were immunoprecipitated with anti-PTBP3 (lower panel). The negative control (Ctr) only has the same amount of protein extracts.

To determine whether PTBP3 can regulate the expression of Id1 isomers through alternative splicing, we overexpressed or silenced PTBP3 in MKN45 and HGC27 cells. After transfection of pcDNA (PTBP3) vector or siRNA (PTBP3), the expression of Id1a and Id1b in MKN45 and HGC27 cells were detected. Our results showed that PTBP3 overexpression upregulated Id1a expression, but downregulated Id1b expression. Conversely, PTBP3 silencing downregulated Id1a expression but upregulated Id1b expression (Figure 3A). Importantly, a xenotransplantation model was used. We found the tumors exhibited higher Id1b expression which from PTBP3 knockdown mice than those from control mice (Figure 3B). PTBP3 is an alternative splicing factor, which plays an alternative splicing role by binding to mRNA. RIP assays indicated that PTBP3 could combine with Id1 mRNA (Figure 3C). This suggested that PTBP3 may upregulate Id1a expression and downregulate Id1b expression through alternative splicing.

Our previous study suggested that PTBP3 can bind to the CU-rich region of the CAV1 intron to regulate the expression of CAV1 splicing isomers (23). To investigate whether PTBP3 also can regulate the expression of Id1 splicing isomers by binding to the CU-rich region of Id1 intron, We performed UV cross-linking experiment for further examination. Radiolabeled intron of Id1 RNA which contains wild type fragment (WT, contains CCUU rich elements) or a CU mutation fragment (MU, contains CCCC elements) were mixed with protein extracts, then, sequence-specific binding of protein was tested by UV cross-linking, and the radiolabeled RNA crosslinked to the proteins was visualized (Figure 3D upper panel). As shown in Figure 3D, the band indicating RNA-protein interaction was present in both the WT and MU groups, but the optical density in the WT group was higher than that observed for the MU group. This finding suggests that PTBP3 can interact with the WT Id1 gene but not with the MU Id1 gene. Our data indicate that PTBP3 upregulates Id1a expression and downregulates Id1b expression through binding to the CU-rich region of Id1 intron.

### The Affinity of Id1b for Hes1 Is More Potent Than That of Id1a

**Figure 4:**
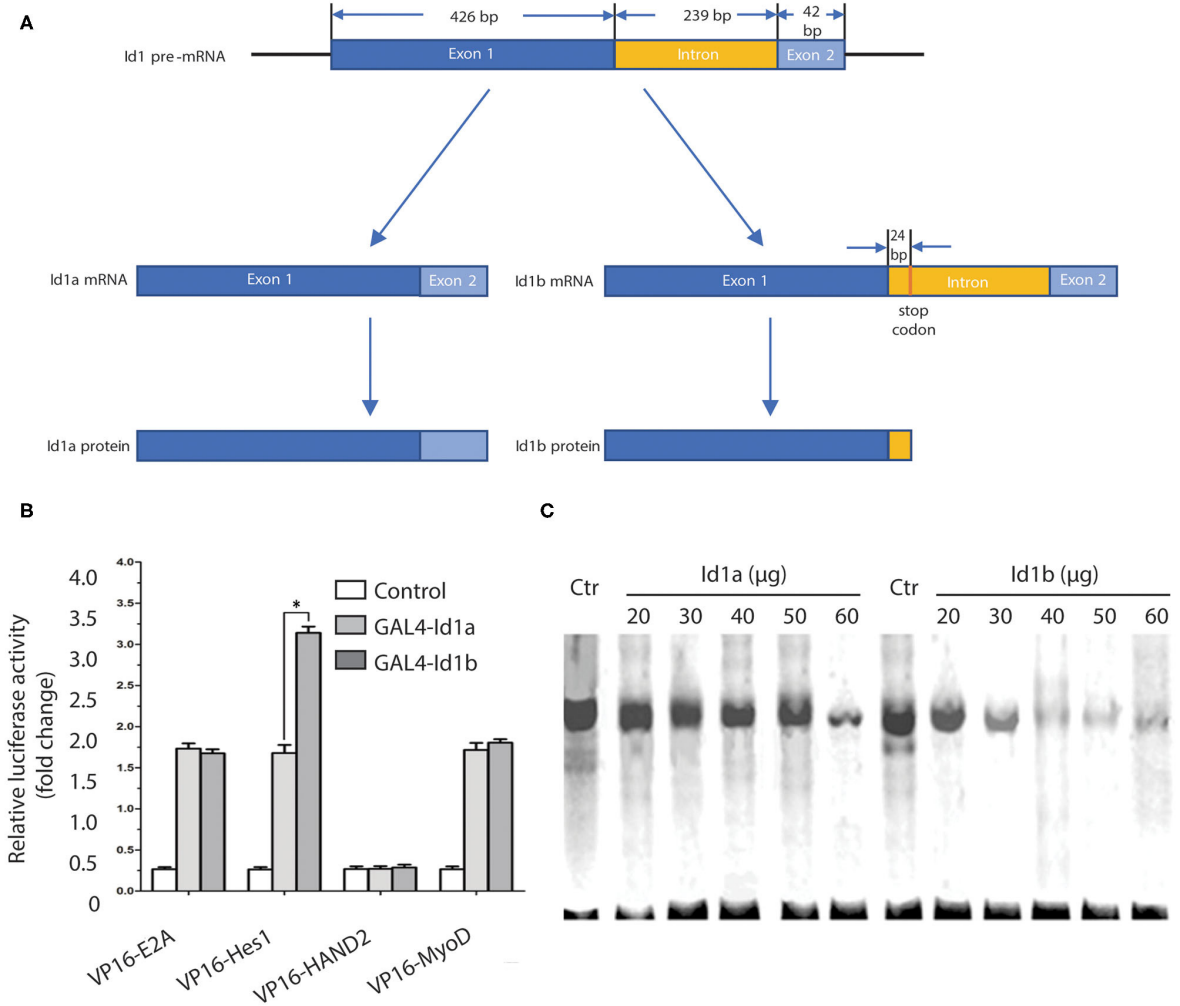
The affinity of Id1b for Hes1 is more potent than that of Id1a. **(A)** The Id1 coding sequence consists of two exons: the 5′ exon of 426 bp, including the HLH region, and the 3′ exon of 42 bp. These exons are separated by an intron of 239 bp. From the sequence of the introns, Id1b is generated by skipping the 5′ splice donor signal following the first exon. This skipping adds 24 bp to the translatable sequence. **(B)** Mammalian two-hybrid analysis of the ability of Id1a or Id1b to dimerize with bHLH transcription factors. Cells were transfected with the indicated plasmids and assayed for luciferase production at 48 h post-transfection. The value is normalized to Renilla luciferase activity. All error bars are mean ± S.D., *n* = 3. *P < 0.05, compared with GAL4-Id1a. **(C)** Electrophoretic mobility shift assays to identify differential Id1a and Id1b protein interaction. The assays were performed with increasing amounts (20, 30, 40, 50, 60 μg) of cell extracts from Id1a- or Id1b-transfected cells, then mixed with a constant amount cell extracts from Hes1-transfected cells. The same amount of cell extracts from untransfected cells (Ctr) was used as a negative control.

The Id1 coding sequence consists of two exons: the 5′ exon of 426 bp, including the HLH region, and the 3′ exon of 42 bp. These exons are separated by an intron of 239 bp. From the sequence of the introns, Id1b is generated by skipping the 5′ splice donor signal following the first exon. This skipping adds 24 bp to the translatable sequence (Figure 4A). These 24 bp encode the 7 C-terminal amino acids, which are unique to Id1b, followed by a stop codon. Therefore, the Id1a and Id1b proteins are identical in the amino acids encoded by the first exon, which includes the HLH domain. Id1a and Id1b are different only at the extreme C-terminus: splicing produces Id1a, which contains 13 amino acids encoded by the second exon, while failing to splice to produce Id1b, which contains 7 different C-terminal amino acids from the intron (18).

To investigate possible differences in the functions of Id1a and Id1b, the two-hybrid analysis was employed to examine the binding activity of each to E2A, Hes1, HAND2, and MyoD. The pACT plasmids expressing the bHLH transcription factor and VP16 fusion protein and pBIND plasmids expressing Id1a or Id1b and the GAL4 fusion protein were prepared. Measurements of the luciferase activity following transfection revealed that no changes with binding of GAL4-Id1a or GAL4-Id1b to VP16-E2A, VP16-HAND2, and VP16-MyoD. However, the binding of GAL4-Id1b to *Hes1* significantly increased luciferase activity as compared to the binding of GAL4-Id1a to *Hes1* (*P* < 0.05, independent sample *t*-test). This indicates that Id1a and Id1b have distinct affinities to Hes1 only; the affinity of Id1b is more potent than that of Id1a (Figure 4B).

To further confirm the difference in the binding activity of Id1a and Id1b to Hes1, electrophoretic mobility shift assays (EMSAs) were employed. A ^32^P-labeled oligonucleotide probe containing an N-box was mixed with the extract of Hes1-transfected cells and then with an extract of Id1a- or Id1b-transfected cells. The results showed that the binding of Hes1 to the oligonucleotide containing N-box was reduced gradually with an increase in the concentration of Id1a or Id1b (20, 30, 40, 50, and 60 μg). After mixing with the extract of Id1b-transfected cells, the binding of Hes1 to N-box was reduced dramatically. Thus, both Id1a and Id1b bind Hes1, which inhibits the binding of Hes1 to the N-box in a dose-dependent manner. As compared to Id1a, Id1b has a more potent interaction with Hes1 (Figure 4C).

### Hes1 Interacts With the N-Box of the *PTBP3* Promoter to Inhibit Its Activity but did not Affect PTBP3 Protein Expression

**Figure 5:**
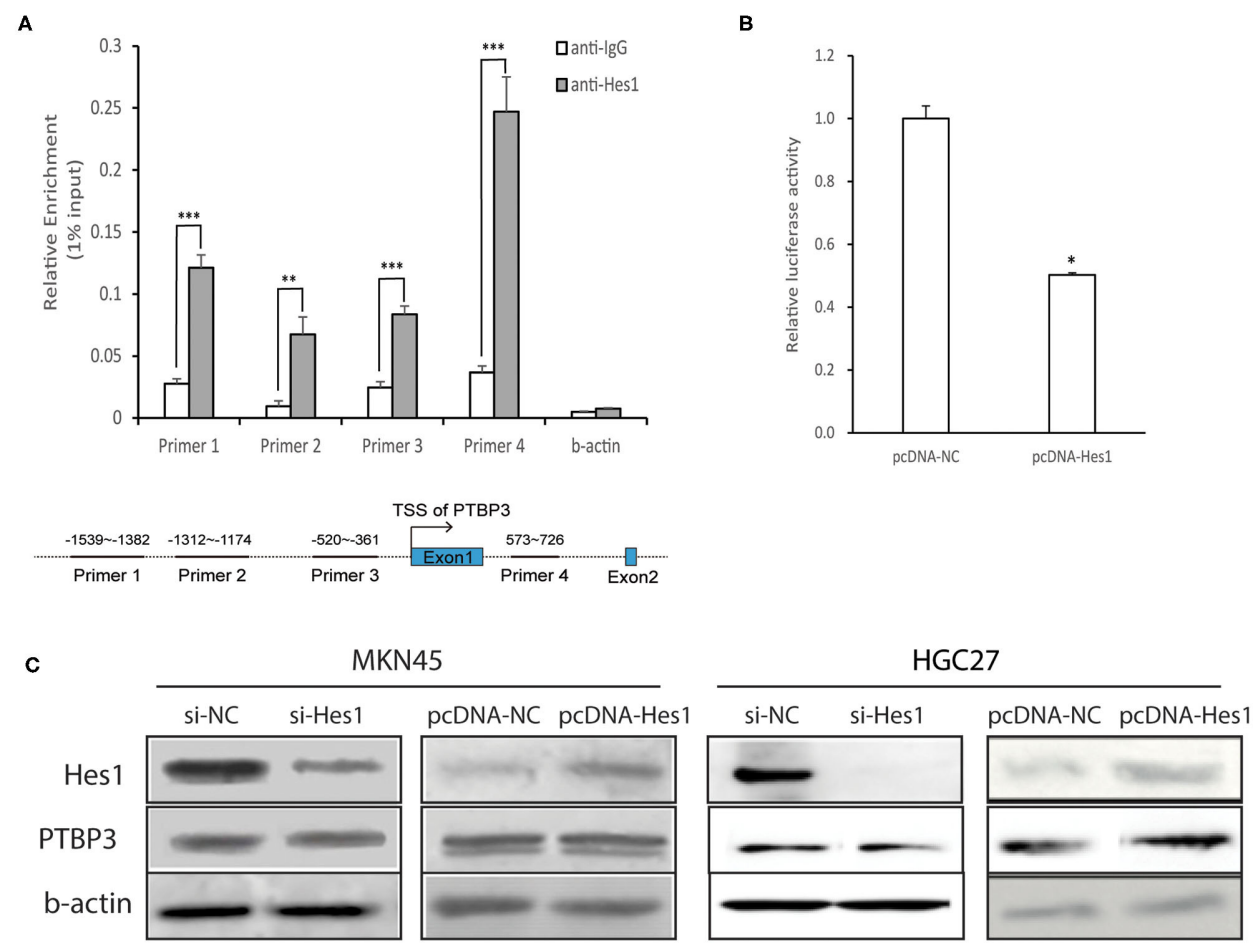
Hes1 represses the activity of the PTBP3 promoter but did not affect PTBP3 protein expression. **(A)** MKN45 cells were cultured and lysates were subjected to ChIP assay. Four paired primers were designed near the predicted binding sites of Hes1. The ChIP assays verified the association of Hes1 and the promoter of *PTBP3* gene. The results are expressed as the mean ± S.D. of three independent experiments. **P < 0.01, ***P < 0.001 vs. anti-IgG. **(B)** The 5′-upstream promoter region (1,098 bp) was inserted into the pGL3-basic plasmid. Reporter activity was also analyzed after transfection of pcDNA (Hes1) into gastric cancer cells. All error bars are mean ± S.D., *n* = 3. *P < 0.05. **(C)** Cells in Si-NC group were transfected with non-targeting siRNA as a negative control. In Si-Hes1 group, cells were treated with Hes1 siRNA. The pcDNA-NC group refers to cells transfected with a blank vector. The pcDNA-Hes1 group refers to cells transfected with Hes1-overexpressing plasmids. After 48 h following transfection, total proteins were extracted, and Hes1 and PTBP3 protein expression were measured. Hes1 expression has no influence on PTBP3 expression in MKN45 and HGC27 cells.

Hes1 is an important transcriptional repressor and can bind to the N-box to inhibit the expression of target genes (24, 25). Analysis of the *PTBP3* promoter showed that it contains a typical N-box sequence (5′-CACAAG-3′). To investigate whether Hes1 combined with *PTBP3*, we performed ChIP assays with four pairs of *PTBP3* primers that designed near the predicted binding sites of Hes1. We found a strong binding of Hes1 on *PTBP3* promoter. The ChIP assays verified the association of Hes1 and the promoter of *PTBP3* (Figure 5A).

To explore the effect of Hes1 on the activity of the *PTBP3* promoter, the *PTBP3* promoter was inserted into the pGL3-basic vector, which together with a Hes1 expression plasmid pcDNA (Hes1) was transfected into MKN45 cells. MKN45 cells transfected with pGL3-basic alone served as a control. Dual luciferase reporter gene assays showed that the luciferase activity was significantly lower when Hes1 was overexpressed than in the control group (*P* < 0.05, independent sample *t*-test). This suggested that Hes1 overexpression inhibits the activity of the *PTBP3* promoter (Figure 5B).

To further investigate the influence of Hes1 on PTBP3 expression, siRNA (Hes1) was used to silence Hes1 expression in MKN45 and HGC27 cells, and PTBP3 protein expression was detected by Western blot assays. As shown in Figure 5C, the expression of PTBP3 protein remained unchanged after Hes1 silencing. In addition, Hes1 overexpression failed to alter the expression of PTBP3. These results indicated that Hes1 expression has no influence on the expression of PTBP3 in gastric cancer cells.

## Discussion

Gastric cancer is the third leading cause of cancer-related death worldwide. Gene abnormalities involved in the regulation of cell differentiation and proliferation play an important role in the development of gastric cancer.

Alternative splicing, a post-transcriptional modification, is an important mechanism for controlling gene expression. Alternative splicing and the resultant protein isoforms with distinct functions are key mechanisms underlying the functional diversity of the proteome (26). Disorders of alternative splicing play an essential role in the pathogenesis of cancers.

The polypyrimidine tract binding protein 1 (PTBP1) is an extensively studied member of the hnRNP protein family (27). PTBP1 has two paralogue proteins: nPTB/PTBP2 (neural PTB) and PTBP3 (polypyrimidine tract binding protein 3). PTBP2 was first identified in neurons (28), but PTBP3 was first found in hematopoietic cells (29). In recent years, there is evidence showing that PTBP2 and PTBP3 are also expressed in many other tissues (30, 31). PTBP1, PTBP2, and PTBP3 proteins share >70% amino acid sequence identity, display similar protein structure and have four RNA recognition motifs (RRM) (7). The molecular function and biological effects of PTBP3 are less understood as compared to PTBP1 and PTBP2 (32).

Initially, it was found that PTBP3 overexpression inhibited NaBU-induced differentiation of leukemic cells (k562 cells). Therefore, PTBP3 was initially named regulator of differentiation 1 (ROD1) (29). We found that the expression of PTBP3 was higher in gastric cancer than in normal gastric mucosa, inhibition of PTBP3 could induce apoptosis of gastric cancer cells and cell cycle arrest (33). Our previous study also indicated that after PTBP3 silencing in gastric cancer cells, the cells showed the characteristics of differentiation, the nucleus/cytoplasmic ratio decreased, the nucleus became regular, and the nucleolus decreased. In addition, the levels of serum LDH and ALP decreased significantly. The growth of tumor xenografts was inhibited when PTBP3 gene was silenced. The results suggested that PTBP3 may have an effect on the proliferation and differentiation of gastric cancer cells (8). These findings suggest that PTBP3 plays a key role in the regulation of gastric cancer cell differentiation, but the specific mechanism is still unclear.

In the present study, we found that PTBP3 was upregulated in the poorly differentiated gastric cancer tissues. Patients with high levels of PTBP3 expression had significantly shorter survival than those with low PTBP3 expression. NaBU can induce the differentiation of many types of cancer cells and has been regarded as a widely accepted differentiation-inducing reagent (34). MKN45 cells were induced to differentiate with NaBU and the expressions of Id1a, Id1b, PTBP3 and Hes1 (proteins closely related to the differentiation of gastric cancer cells) were detected. In our study, the protein expression of Id1a decreased over time, accompanied by a gradual increase of Id1b. The changes in mRNA expression of Id1a and Id1b were similar to those of their proteins, indicating that Id1a expression declines, but Id1b expression increases with the progression of cell differentiation. Previous studies have also found that the protein expression of Id1a in gastric cancer cells was downregulated with cell differentiation, but the mRNA expression remained unchanged (17). This might be ascribed to the design of different primers for Id1a and Id1b. This also implies that there is a post-translational regulation of *Id1*. In addition, our results also showed that the protein expression of PTBP3 was reduced gradually over time after NaBU treatment, suggesting that it is related to the regulation of gastric cancer differentiation. Moreover, the protein expression of Hes1 progressively decreased over time, which is consistent with Hes1-induced inhibition of cancer cell differentiation (11). The regulatory effects of Hes1 might be mediated through the Notch signaling pathway (35).

The carcinogenesis of cells is closely related to abnormal proliferation and differentiation of these cells. Id proteins are important inhibitors of cellular differentiation and were first cloned from the cDNA library of murine erythroleukemia (MEL) cells. They share a helix-loop-helix (HLH) structure (36). A total of four Id proteins have been identified: Id1, Id2, Id3, and Id4 (37, 38). Id1 is widely expressed in a variety of human cancers and inhibits cell differentiation, promotes the occurrence, and development of cancers (39–41).

In this study, RNA interference and gene overexpression were employed to confirm that PTBP3, a protein involved in the alternative splicing, could regulate the expression of Id1a and Id1b. We showed that PTBP3 positively regulates the expression of Id1a, but negatively regulates Id1b expression. Further cross-linking analyses confirmed that PTBP3 could bind to the CU-rich region in the intron of the *Id1* gene to upregulate Id1a expression and downregulate Id1b expression. Two-hybrid analysis and EMSAs revealed that Id1a and Id1b had distinct affinities to the Hes1 protein; the binding activity of Id1b to Hes1 is more potent than that of Id1a. Id1a and Id1b have different structures at their C-termini (42), which might contribute to the different affinities for Hes1. Thus, PTBP3 overexpression may upregulate Id1a expression and downregulate Id1b expression to attenuate the inhibition of Hes1 activity, which indirectly increases the activity of Hes1, leading to the inhibition of cancer cell differentiation. We confirmed that Hes1 could bind the N-box sequence in the *PTBP3* promoter and inhibit its activity. This implies that Hes1 may repress the transcription of PTBP3. However, after silencing or overexpressing Hes1 in MKN45 and HGC27 cells, the protein expression of PTBP3 remained unchanged. PTBP3 expression inversely correlated with Id1b expression and had no correlation with Hes1 in gastric cancer tissues.

**Figure 6:**
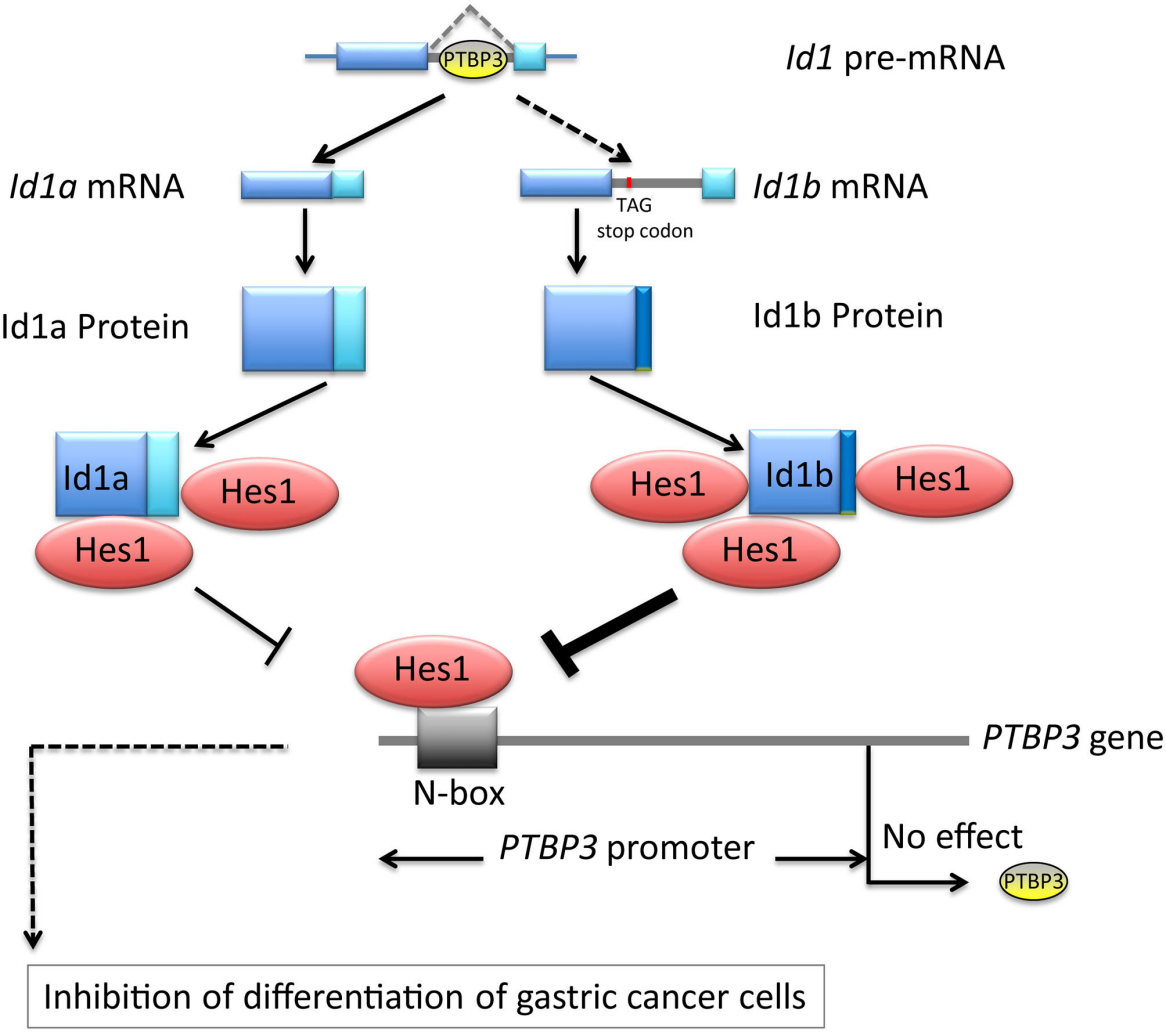
Schematic depicting PTBP3 induced inhibition of differentiation of gastric cancer cells through alternative splicing of Id1. The loss of feedback regulation among Id1a, Id1b, and Hes1 may be a major cause of malignant transformation.

Our results indicated that PTBP3 can regulate the expression of Id1a and Id1b isoforms via alternative splicing. Id1a and Id1b may differentially bind to Hes1 to inhibit Hes1 activity, which may be related to PTBP3-induced inhibition of cellular differentiation. Although Hes1 inhibited the activity of the *PTBP3* promoter, it failed to impact the protein expression of PTBP3, showing the complexity of gene expression regulation in gastric cancer cells. Thus, in gastric cancer, the loss of feedback regulation among Id1a, Id1b, and Hes1 may be a major cause of malignant transformation (Figure 6). Our findings are helpful to understand the relationship between alternative splicing and the occurrence and development of gastric cancer.

## Data Availability Statement

The datasets generated for this study are available on request to the corresponding author.

## Ethics Statement

The animal study was reviewed and approved by the Animal Ethics Committee of Longhua Hospital.

## Author Contributions

AZ, XL, and JL participated in the conception and design of the study. BC and WC performed the experiments. XM, LY, and XG interpreted the data produced and edited the drafts of the manuscript. All authors read and approved the final manuscript.

## Conflict of Interest

The authors declare that the research was conducted in the absence of any commercial or financial relationships that could be construed as a potential conflict of interest.

## References

[1] Wang ET Sandberg R Luo S Khrebtukova I Zhang L Mayr C. Alternative isoform regulation in human tissue transcriptomes. Nature. (2008) 456:470–6. 10.1038/nature07509 18978772 PMC2593745

[2] Hagen RM Ladomery MR. Role of splice variants in the metastatic progression of prostate cancer. Biochem Soc Transact. (2012) 40:870–4. 10.1042/BST20120026 22817750

[3] David CJ Manley JL. Alternative pre-mRNA splicing regulation in cancer: pathways and programs unhinged. Genes Develop. (2010) 24:2343–64. 10.1101/gad.1973010 21041405 PMC2964746

[4] Oltean S Bates DO. Hallmarks of alternative splicing in cancer. Oncogene. (2014) 33:5311–8. 10.1038/onc.2013.533 24336324

[5] Venables JP Klinck R Bramard A Inkel L Dufresnemartin Gv Koh CS. Identification of alternative splicing markers for breast cancer. Cancer Res. (2008) 68:9525. 10.1158/0008-5472.CAN-08-1769 19010929

[6] Graveley BR. Sorting out the complexity of SR protein functions. RNA. (2000) 6:1197–211. 10.1017/S1355838200000960 10999598 PMC1369994

[7] Spellman R Llorian M Smith CW. Crossregulation and functional redundancy between the splicing regulator PTB and its paralogs nPTB and ROD1. Mol Cell. (2007) 27:420–34. 10.1016/j.molcel.2007.06.016 17679092 PMC1940037

[8] Chen B Zhao AG Shao J Mu XY Jiang L Liu JW. The effects of PTBP3 silencing on the proliferation and differentiation of MKN45 human gastric cancer cells. Life Sci. (2014) 114:29–35. 10.1016/j.lfs.2014.07.038 25119103

[9] Murre C Bain G van Dijk MA Engel I Furnari BA Massari ME. Structure and function of helix-loop-helix proteins. Biochim Biophys Acta. (1994) 1218:129. 10.1016/0167-4781(94)90001-9 8018712

[10] Sekine A Akiyama Y Yanagihara K Yuasa Y. Hath1 up-regulates gastric mucin gene expression in gastric cells. Biochem Biophys Res Commun. (2006) 344:1166. 10.1016/j.bbrc.2006.03.238 16647036

[11] Sang L Roberts JM Coller HA. Hijacking HES1: tumors co-opt the anti-differentiation strategies of quiescent cells. Trends Mol Med. (2010) 16:17. 10.1016/j.molmed.2009.11.001 20022559 PMC2864914

[12] Sikder HA Devlin MK Dunlap S Ryu B Alani MR. Id proteins in cell growth and tumorigenesis. Cancer Cell. (2003) 3:525–30. 10.1016/S1535-6108(03)00141-7 12842081

[13] Schmidt M Asirvatham AJ Chaudhary J. Inhibitor of differentiation 1 (Id1) promotes cell survival and proliferation of prostate epithelial cells. Cell Mol Biol Lett. (2010) 15:272. 10.2478/s11658-010-0007-3 20186495 PMC6276005

[14] Meyer KB Skogberg M Margenfeld C Ireland J Pettersson S. Repression of the immunoglobulin heavy chain 3' enhancer by helix-loop-helix protein Id3 via a functionally important E47/E12 binding site: implications for developmental control of enhancer function. Eur J Immunol. (1995) 25:1770–7. 10.1002/eji.1830250643 7615006

[15] Wilson RB Kiledjian M Shen CP Benezra R Zwollo P Dymecki SM. Repression of immunoglobulin enhancers by the helix-loop-helix protein Id: implications for B-lymphoid-cell development. Mol Cell Biol. (1991) 11:6185. 10.1128/MCB.11.12.6185 1944284 PMC361801

[16] Yang HY Liu HL Liu GY Zhu H Meng QW Qu LD. Expression and prognostic values of Id-1 and Id-3 in gastric adenocarcinoma. J Surg Res. (2011) 167:258. 10.1016/j.jss.2009.08.006 20080245

[17] Han S Gou C Hong L Liu J ZheyiHan Liu C. Expression and significances of Id1 helix-loop-helix protein overexpression in gastric cancer. Cancer Lett. (2004) 216:63–71. 10.1016/j.canlet.2004.07.035 15575081

[18] Nehlin JO Hara E Kuo WL Collins C Campisi J. Genomic organization, sequence, and chromosomal localization of the human helix-loop-helix Id1 gene. Biochem Biophys Res Commun. (1997) 231:628–34. 10.1006/bbrc.1997.6152 9070860

[19] Nguewa P Manrique I Redrado M Parrondo R Perezstable C Calvo A. Id-1B, an alternatively spliced isoform of the inhibitor of differentiation-1, impairs cancer cell malignancy through inhibition of proliferation and angiogenesis. Curr Mol Med. (2014) 14:151–62. 10.2174/1566524013666131203100643 24295493

[20] Manrique I Nguewa P Bleau AM Nistal E Lopez I Villalba M. The inhibitor of differentiation isoform Id1b, generated by alternative splicing, maintains cell quiescence and confers self-renewal and cancer stem cell-like properties. Cancer Lett. (2015) 356:899. 10.1016/j.canlet.2014.10.035 25449776

[21] Xu W Liu J Li C Wu HZ Liu YW. Kaempferol-7-O-beta-D-glucoside (KG) isolated from Smilax china L. rhizome induces G2/M phase arrest and apoptosis on HeLa cells in a p53-independent manner. Cancer Lett. (2008) 264:229–40. 10.1016/j.canlet.2008.01.044 18343026

[22] Bai Z Zhang Z Ye Y Wang S. Sodium butyrate induces differentiation of gastric cancer cells to intestinal cells via the PTEN/phosphoinositide 3-kinase pathway. Cell Biol Int. (2010) 34:1141–5. 10.1042/CBI20090481 20718712

[23] Liang X Chen W Shi H Gu X Li Y Qi Y. PTBP3 contributes to the metastasis of gastric cancer by mediating CAV1 alternative splicing. Cell Death Dis. (2018) 9:569. 10.1038/s41419-018-0608-8 29752441 PMC5948206

[24] Iso T Sartorelli V Chung G Shichinohe T Kedes L Hamamori Y. HERP, a new primary target of Notch regulated by ligand binding. Mol Cell Biol. (2001) 21:6071. 10.1128/MCB.21.17.6071-6079.2001 11486044 PMC87324

[25] Takebayashi K Sasai Y Sakai Y Watanabe T Nakanishi S Kageyama R. Structure, chromosomal locus, and promoter analysis of the gene encoding the mouse helix-loop-helix factor HES-1. negative autoregulation through the multiple N box elements. J Biol Chem. (1994) 269:5150–6. 7906273

[26] Maniatis T Tasic B. Alternative pre-mRNA splicing and proteome expansion in metazoans. Nature. (2002) 418:236–43. 10.1038/418236a 12110900

[27] Wagner EJ Garcia-Blanco MA. Polypyrimidine tract binding protein antagonizes exon definition. Mol Cell Biol. (2001) 21:3281–8. 10.1128/MCB.21.10.3281-3288.2001 11313454 PMC100250

[28] Kikuchi T Ichikawa M Arai J Tateiwa H Fu L Higuchi K. Molecular cloning and characterization of a new neuron-specific homologue of rat polypyrimidine tract binding protein. J Biochem. (2000) 128:811. 10.1093/oxfordjournals.jbchem.a022819 11056394

[29] Yamamoto H Tsukahara K Kanaoka Y Jinno S Okayama H. Isolation of a mammalian homologue of a fission yeast differentiation regulator. Mol Cell Biol. (1999) 19:3829. 10.1128/MCB.19.5.3829 10207106 PMC84229

[30] Markovtsov V Nikolic JM Goldman JA Turck CW Chou MY Black DL. Cooperative assembly of an hnRNP complex induced by a tissue-specific homolog of polypyrimidine tract binding protein. Mol Cell Biol. (2000) 20:7463. 10.1128/MCB.20.20.7463-7479.2000 11003644 PMC86300

[31] Maud N Yann A Serge H. Expression analysis of the polypyrimidine tract binding protein (PTBP1) and its paralogs PTBP2 and PTBP3 during Xenopus tropicalis embryogenesis. Int J Develop Biol. (2012) 56:747–53. 10.1387/ijdb.120017sh 23124965

[32] Tan LY Whitfield P Llorian M Monzoncasanova E Diazmunoz MD Turner M. Generation of functionally distinct isoforms of PTBP3 by alternative splicing and translation initiation. Nucleic Acids Res. (2015) 43:5586. 10.1093/nar/gkv429 25940628 PMC4477659

[33] Liang X Shi H Yang L Qiu C Lin S Qi Y. Inhibition of polypyrimidine tract-binding protein 3 induces apoptosis and cell cycle arrest, and enhances the cytotoxicity of 5- fluorouracil in gastric cancer cells. Br J Cancer. (2017) 116:903. 10.1038/bjc.2017.32 28222070 PMC5379144

[34] Wang A Zeng R Huang H. Retinoic acid and sodium butyrate as cell cycle regulators in the treatment of oral squamous carcinoma cells. Oncol Res. (2008) 17:175–82. 10.3727/096504008785114129 18773862

[35] Jenkins D. Hedgehog signalling: emerging evidence for non-canonical pathways. Cell Signal. (2009) 21:1023–34. 10.1016/j.cellsig.2009.01.033 19399989

[36] Benezra R Davis RL Lockshon D Turner DL Weintraub H. The protein Id: a negative regulator of helix-loop-helix DNA binding proteins. Cell. (1990) 61:49–59. 10.1016/0092-8674(90)90214-Y 2156629

[37] Massari ME Murre C. Helix-loop-helix proteins: regulators of transcription in eucaryotic organisms. Mol Cell Biol. (2000) 20:429. 10.1128/MCB.20.2.429-440.2000 10611221 PMC85097

[38] Sun XH Copeland NG Jenkins NA Baltimore D. Id proteins Id1 and Id2 selectively inhibit DNA binding by one class of helix-loop-helix proteins. Mol Cell Biol. (1991) 11:5603–11. 10.1128/MCB.11.11.5603 1922066 PMC361931

[39] Maw MK Fujimoto J Tamaya T. Expression of the inhibitor of DNA-binding (ID)-1 protein as an angiogenic mediator in tumour advancement of uterine cervical cancers. Br J Cancer. (2008) 99:1557. 10.1038/sj.bjc.6604722 19002177 PMC2584935

[40] Forootan SS Wong YC Dodson A Wang X Lin K Smith PH. Increased Id-1 expression is significantly associated with poor survival of patients with prostate cancer. Hum Pathol. (2007) 38:1321–9. 10.1016/j.humpath.2007.02.011 17599389

[41] Ding Y Wang GM Wong YC Li X Na Y Zhang X. Significance of Id-1 up-regulation and its association with EGFR in bladder cancer cell invasion. Int J Oncol. (2006) 28:847–54. 10.3892/ijo.28.4.847 16525633

[42] Hara E Hall M Peters G. Cdk2-dependent phosphorylation of Id2 modulates activity of E2A-related transcription factors. EMBO J. (1997) 16:332–42. 10.1093/emboj/16.2.332 9029153 PMC1169639

